# Enhancement of 5-fluorouracil-induced cytotoxicity by leucovorin in 5-fluorouracil-resistant gastric cancer cells with upregulated expression of thymidylate synthase

**DOI:** 10.1007/s10120-013-0249-7

**Published:** 2013-03-15

**Authors:** Ayako Nakamura, Go Nakajima, Ryuji Okuyama, Hidekazu Kuramochi, Yurin Kondoh, Toshinori Kanemura, Teiji Takechi, Masakazu Yamamoto, Kazuhiko Hayashi

**Affiliations:** 1 Field of Chemotherapy on Digestive Organs Division of Gastrointestinal Surgery, Tokyo Women’s Medical University Graduate School of Medicine, 8-1 Kawada-cho, Shinjuku-ku, Tokyo, 162-8666 Japan; 2 Department of Chemotherapy and Palliative Care, Tokyo Women’s Medical University, 8-1 Kawada-cho, Shinjuku-ku, Tokyo, 162-8666 Japan; 3 Oncology Medical Affairs Department, Taiho Pharmaceutical Co., Ltd, 1-2-4 Uchikanda, Chiyoda-ku, Tokyo, 101-0047 Japan; 4 Department of Surgery, Institute of Gastroenterology, Tokyo Women’s Medical University, 8-1 Kawada-cho, Shinjuku-ku, Tokyo, 162-8666 Japan; 5 Laboratory for Oncology Medication Management and Development, Taiho Pharmaceutical Co., Ltd, 1-2-4 Uchikanda, Chiyoda-ku, Tokyo, 101-0047 Japan

**Keywords:** Gastric cancer, 5-Fluorouracil, Leucovorin, Acquired resistance, Thymidylate synthase

## Abstract

**Background:**

Elucidation of the mechanisms by which gastric cancer cells acquire resistance to 5-fluorouracil (5FU) may provide important clues to the development of effective chemotherapy for 5FU-resistant gastric cancer

**Methods:**

Four 5FU-resistant cell lines (MKN45/5FU, MKN74/5FU, NCI-N87/5FU, and KATOIII/5FU) were established by continuous exposure of the cells to progressively increasing concentrations of 5FU for about 1 year. Then, mRNA expression levels of four genes associated with 5FU metabolism, i.e., thymidylate synthase (TS), dihydropyrimidine dehydrogenase, thymidine phosphorylase, and orotate phosphoribosyltransferase, were quantitatively evaluated by real-time reverse transcriptase-polymerase chain reaction. In addition, TS protein expression was measured by Western blot analysis.

**Results:**

As compared with the parent cell lines, the 5FU-resistant cell lines showed 3.8- to 11.6-fold higher resistance to 5FU, as well as 1.9- to 3.5-fold higher TS mRNA expression and 1.6- to 7.1-fold higher TS protein expression. In contrast, the expressions of other genes did not differ significantly among the cell lines. The cytotoxicity of 5FU was enhanced 2.3- to 2.8 fold by leucovorin (LV) against three of the four 5FU-resistant cell lines.

**Conclusions:**

Collectively, LV enhanced the cytotoxicity of 5FU not only against the parent gastric cancer cell lines, but also against the 5FU-resistant cell lines, even those with elevated TS expression levels. These results suggest that clinical studies of a combination of 5FU and LV are warranted in patients who have recurrent gastric cancer after 5FU-based therapy.

## Introduction

Worldwide, gastric cancer ranks third among males and fifth among females among all causes of death from cancer [1]. Significant survival benefits of 5-fluorouracil (5FU)-based chemotherapy have been reported in patients with metastatic gastric cancer as well as those who have undergone surgery [2–5]. Although such regimens have improved response, many patients have recurrence after several courses of 5FU-based chemotherapy. The inherent or acquired resistant of certain tumors to 5FU therapy is thus a major clinical problem, but the molecular mechanisms underlying the development of 5FU chemoresistance in patients with cancer remain poorly understood.

Thymidylate synthase (TS) has been recognized as the rate-limiting enzyme in de novo pyrimidine biosynthesis. TS is inhibited by 5-fluoro-2′-deoxyuridinemonophosphate (FdUMP) formed from 5-FU in the presence of the folate co-factor, 5,10-methylenetetrahydrofolate (CH_2_FH_4_), leading to inhibition of DNA synthesis [2, 6]. High expression of TS is induced by continuous exposure of cancer cell lines to 5FU [7]. TS expression influences response to 5FU-based chemotherapy and survival in patients with gastric cancer [8, 9]. Several clinical studies have examined the relationships of clinical response and survival to the tumor expression levels of TS and other enzymes involved in 5FU metabolism, such as dihydropyrimidine dehydrogenase (DPD), thymidine phosphorylase (TP), and orotate phosphoribosyltransferase (OPRT), in patients with cancer who received 5FU-based chemotherapy [9–12].

It is reported that 5FU-resistant cell lines were established by repeatedly exposing colorectal cancer cell lines to 5FU. These 5FU-resistant cell lines show increased TS mRNA expression, protein expression, and activity as compared with their respective parent cells, as demonstrated by in vitro and vivo assays [13, 14]. The findings of these studies suggest that acquisition of resistance to 5FU is related to increased TS expression. Furthermore, concurrent treatment with LV has been shown to enhance the antitumor activity of tegafur–uracil (UFT) against 5FU-resistant colorectal tumor xenografts with increased TS expression [15], suggesting that 5FU-based therapy including LV may overcome resistance to 5FU caused by increased TS expression. However, these results were derived from colon cancer cells, and studies evaluating the effects of LV in 5FU-resistant gastric cancer cell lines remain scant. Recent biological studies of tumors revealed that gastric cancer is relatively heterogeneous, with a less stable genome than that of colon cancer [16]. Therefore, it is important to confirm the effects of LV on 5FU-resistant cell lines of gastric cancer.

In this study, we established gastric cancer cell lines that were resistant to 5FU by continuous, stepwise escalation of 5FU. We then analyzed the mechanism underlying the development of resistance to 5FU in association with the upregulation of TS mRNA level in 5FU-resistant gastric cells. To our knowledge, this is the first study to assess the enhancement of the cytotoxicity of 5FU by LV in both the parent and 5FU-resistant gastric cancer cell lines.

## Materials and methods

### Gastric cancer cell lines and establishment of 5FU-resistant cell lines

MKN45 was obtained from the Health Science Research Resource Bank (Tokyo, Japan), MKN74 and KATOIII were obtained from RIKEN BRC Cell Bank (Ibaraki, Japan), and NCI-N87 was obtained from the American Type Culture Collection (Rockville, MD, USA). Only KATOIII was derived from primary gastric cancer; the other three cell lines were derived from sites of liver metastasis. Tissue types were classified as diffuse type (MKN45 and KATOIII) or intestinal type (MKN74 and NCI-N87). As for *p53* status, NCI-N87 has mutation, KATOIII has gross deletion, and the other two cell lines were wild type [16–18]. 5FU-resistant cells (MKN45/5FU, MKN74/5FU, NCI-N87/5FU, and KATOIII/5FU) were established from each parent cell line by repeated, continuous (3- to 5-day) exposure of the cell cultures to escalating concentrations of 5FU for about 1 year. Cell lines were maintained in RPMI-1640 (Gibco BRL, Gaithersburg, MD, USA) with 10 % fetal bovine serum (FBS; JRH Biosciences, Lenexa, KS, USA). All cell lines were incubated at 37 °C in a humidified atmosphere of 95 % air and 5 % CO_2_. All cell lines were checked for short tandem repeats (STR) before the study. All experiments were performed using exponentially growing cells.

### Chemicals

The anticancer agent 5-FU was purchased from Wako Pure Chemical Industries (Osaka, Japan), and leucovorin was provided by Taiho Pharmaceutical (Tokyo, Japan).

### Cytotoxicity assay

Resistant cell lines were maintained in drug-free medium for three passage cultures before use. Cell lines were seeded at a density of 1,000 cells per well into 96-well plates and precultured for 24 h. Cell lines were then exposed to various concentrations of 5FU and 10 μM LV for 72 h as described previously [19]. We evaluated the in vitro cytotoxic effects of 5FU with or without LV on the cell lines using 4-[3-(2-methoxy-4-nitrophenyl)-2-(4-nitrophenyl)-2*H*-5-tetrazolio]-l,3-benzene disulfonate sodium salt (WST-8; Dojindo Laboratories, Kumamoto, Japan). The 5FU concentration that inhibited cell growth by 50 % (IC_50_) was calculated from the regression lines. The degree of resistance to 5FU with or without LV was estimated by dividing the IC_50_ of 5FU against the 5FU-resistant cell lines by the IC_50_ of 5FU against the respective parent cell lines.

### Real-time reverse transcriptase-polymerase chain reaction (RT-PCR)

Total RNA in each cell line was isolated using an RNeasy mini kit (Qiagen), as outlined by the manufacturer. After reverse transcription into cDNA using a High Capacity cDNA Archives Kit (Applied Biosystems), the mRNAs of TS, DPD, TP, OPRT, and β-actin, used as an internal reference gene, were determined using a fluorescence-based real-time detection method [ABI Step One System (TaqMan); Applied Biosystems, Foster City, CA, USA]. TaqMan Gene Expression Assays (Applied Biosystems), prevalidated assays that include specific primers and probes for each gene, were used for cDNA quantification of the TS, DPD, TP, and OPRT genes (assay IDs: [TS (TYMS)], Hs00426591_m1; [DPD (DPYD)], Hs00559278_m1; [TP (TYMP)], Hs00157317_m1; [OPRT (UMPS)]). The sequence of the β-actin (ACTB) primers and probe used were as follows: forward, 5′-GAGCGCGGCTACAGCTT-3′; reverse, 5′-TCCTTAATGTCACGCACGATTT-3′; probe, 5′-(FAM) ACCACCACGGCCGAGCGG-3′ [20]. For real-time RT-PCR, TaqMan Universal PCR Master Mix was used (Applied Biosystems). The PCR protocol consisted of 2 min at 50 °C, 10 min at 95 °C, followed by 40 cycles of 30 s at 97 °C and 1 min at 60 °C in the ABI Step One System. Each data point represents the mean of duplicate assays. Relative gene expression was calculated by comparing the difference in cycle threshold values between the gene of interest and the endogenous control (Δ*C*_t_) for target genes and reference genes.

### Western blot analysis

Cytosol of the supernatant from the parent and 5FU-resistant cell lines were homogenized, centrifuged at 105,000 *g* for 60 min, and subjected to Western blot analysis. The cytosol was heated for 10 min at 70 °C and loaded on 4–12 % polyacrylamide gel. After electrophoresis, the proteins were electrically blotted on a polyvinylidene fluoride (PVDF) membrane on ice. The proteins in the PVDF membrane were detected by horseradish peroxidase-conjugated antibody using lumial as substrate. In this experiment, anti-hTS mouse monoclonal antibody, obtained from Taiho Pharmaceutical, and anti-human β-actin antibody (Sigma Chemical, St. Louis, MO, USA) were used as primary antibodies, and anti-mouse IgG was used as secondary antibody.

### Statistical analysis

Statistical analysis was performed using Student’s *t* test with JMP software (SAS, Cary, NC, USA). *P* values <0.05 were considered to indicate statistical significance.

## Results

### Establishment of 5FU-resistant cell lines

**Fig. 1:**
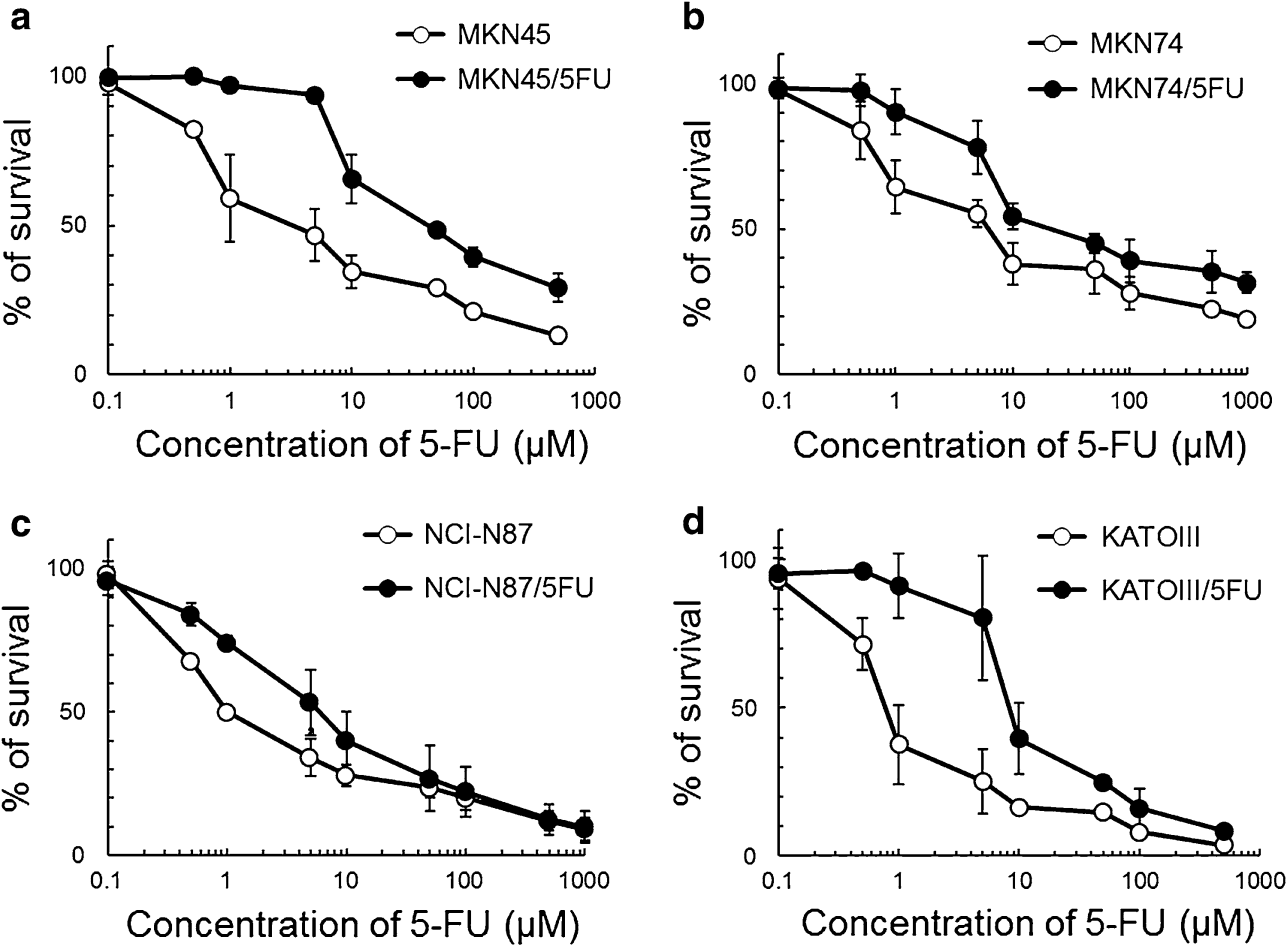
In vitro sensitivity of parent and 5-fluorouracil (5FU)-resistant cell lines to 5FU. Cell lines were cultured with various concentrations of 5FU for 72 h. Each data point represents the mean ± SD (*n* = 3). All 5FU-resistant cell lines were more resistant to 5FU than the parent cell lines. **a** MKN45; **b** MKN74; **c** NCI-N87; **d** KATOIII

The degree of resistance to 5FU was estimated as the ratio of the IC_50_ of each resistant line to that of the respective parent cell line after cells were exposed to various concentrations of 5FU for 4 days. As shown in Fig. 1 and Table 1, each of the resistant lines had acquired high resistance to 5FU, although the degree of resistance varied. IC_50_ of the 5FU-resistant cell lines was 3.8- to 11.6 fold higher than that of the parent cell lines.

**Table 1:** Sensitivity of parent and 5-fluorouracil (5FU)-resistant cell lines to 5FU

Cell line	IC_50_ of 5FU (μM)		Degree of resistance to 5FU
	Parent cell lines	5FU-resistant cell lines	
MKN45	5.1	59.3	11.6
MKN74	9.6	55.4	5.8
NCI-N87	2.3	8.8	3.8
KATOIII	1.2	12.8	10.7

IC_50_ values of 5FU 72 h after treatment (i.e., the 5FU concentration in μM that inhibited cell growth by 50 %)

### mRNA expression levels in 5FU-resistant cell lines

**Fig. 2:**
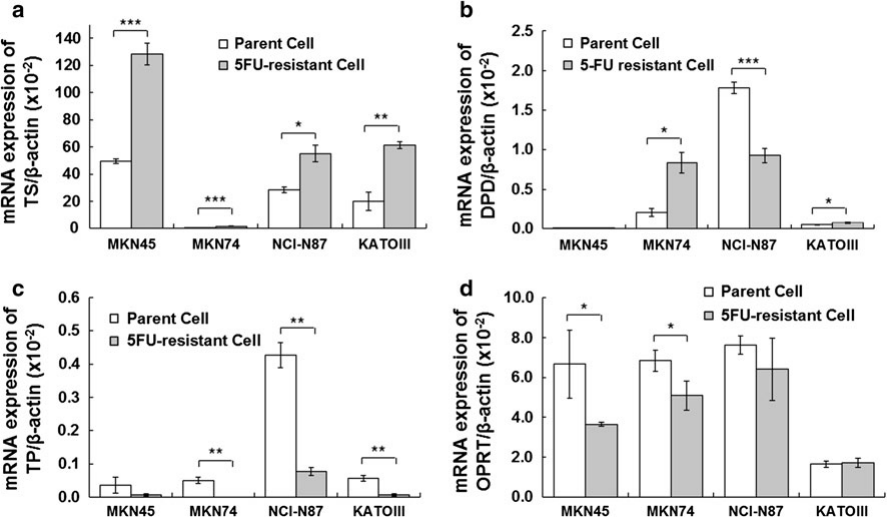
Levels of thymidylate synthase (TS) (**a**), dihydropyrimidine dehydrogenase (DPD) (**b**), thymidine phosphorylase (TP) (**c**), and orotate phosphoribosyltransferase (OPRT) (**d**) mRNAs. The mRNA levels were measured by quantitative real-time RT-PCR and normalized by the level of β-actin mRNA. Each data point represents the mean ± SE of at least duplicate determinations. *P < 0.05, **P < 0.001, ***P < 0.0001 by Student’s *t* test

The mRNA expression levels of TS, DPD, TP, and OPRT were determined by real-time RT-PCR assay. All 5FU-resistant cell lines showed significantly increased mRNA expression of TS, ranging from 1.9- to 3.5 fold higher than that of the parent cell lines. The mRNA expression of TP had decreased in all 5FU-resistant cell lines and was equivalent to <0.2 fold that of the respective parent cell lines. In contrast, the mRNA expression of DPD and OPRT did not differ between the 5FU-resistant cells and the parent cell lines. Only the mRNA levels of TS, which was normalized according to the expression of β-actin, was increased more than 100 fold and were significantly (*p* < 0.05–0.001) higher for 5FU-resistant cell lines compared to parent cell lines (Fig. 2; Table 2). These findings suggested that TS upregulation played a major role in 5FU resistance.

**Table 2:** Ratios of mRNA expression in 5FU-resistant cell lines to that of the respective parent cell lines

Cell line	Ratio of mRNA expression (5FU-resistant/parent)			
	Thymidylate synthase (TS)	Dihydropyrimidine dehydrogenase (DPD)	Thymidine phosphorylase (TP)	Orotate phosphoribosyltransferase (OPRT)
MKN45	2.6	0.9	0.2	0.5
MKN74	3.5	3.9	<0.01	0.7
NCI-N87	1.9	0.5	0.2	0.8
KATOIII	3.0	1.6	0.1	1.0

### Western blot analysis of TS expression

**Fig. 3:**
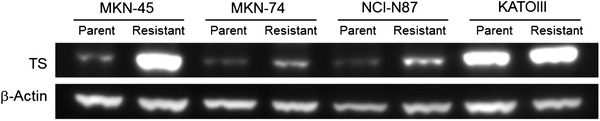
Expression levels of TS and β-actin in parent or 5FU-resistant gastric cancer cell lines on Western blot analysis

To confirm that increased TS expression was associated with acquisition of 5FU resistance, TS protein expression was measured by Western blotting. The ratio of TS to β-actin protein expression in the 5FU-resistant cell lines had increased by 7.1 fold in MKN45, 3.1 fold in MKN74, 3.8 fold in NCI-N87, and 1.6 fold in KATOIII as compared with the respective parent cell lines (Fig. 3).

### Enhancement of 5FU cytotoxicity by LV in parent and 5FU-resistant cells

**Fig. 4:**
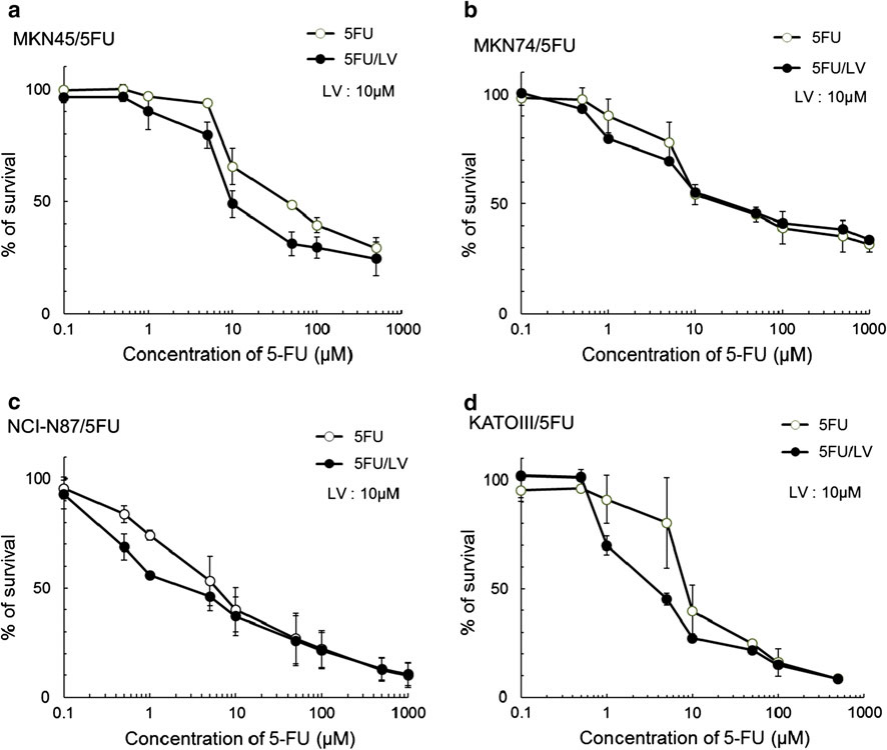
In vitro sensitivity of 5FU-resistant cell lines to 5FU ± LV (leucovorin). Cell lines were cultured with various concentrations of 5FU and 10 μM LV for 72 h. Each data point represents the mean ± SD of triplicate assays. The antitumor activity of 5FU/LV was higher than that of 5FU alone for all 5FU-resistant cell lines. **a** MKN45/5FU; **b** MKN74/5FU; **c** NCI-N87/5FU; **d** KATOIII/5FU

We examined the cytotoxicity of 5FU plus 10 μM LV against each parent cell line by WST-8 assay. LV enhanced the cytotoxicity of 5FU by 2.2–12.3 times against three of the four parent cell lines, excluding MKN74 (Fig. 4; Table 3). LV also enhanced the antitumor activity of 5FU by 2.3–2.8 times against three of the four 5FU-resistant cell lines, excluding MKN74/5FU.

**Table 3:** Antitumor activity of 5FU ± leucovorin (LV) against 5FU-resistant cell lines

Cell line	IC_50_ of 5FU (μM)		Degree of antitumor effect ascribed to LV^a^
	5FU alone	5FU/LV	
MKN45	5.1	2.3	2.2
MKN45/5FU	59.3	21.4	2.8
MKN74	9.6	8.2	1.2
MKN74/5FU	55.4	56.9	1.0
NCI-N87	2.3	0.3	7.4
NCI-N87/5FU	8.8	3.9	2.3
KATOIII	1.2	0.1	12.3
KATOIII/5FU	12.8	4.7	2.7

^a^Ratio of IC_50_ of 5FU alone to IC_50_ of 5FU/LV for each cell line

## Discussion

In this study, we first established four gastric cancer cell lines with acquired resistance to 5FU by continuous and stepwise escalation of 5FU in the culture media. The IC_50_ of the 5FU-resistant cell lines was 3.8- to 11.6 fold higher than that of the parent cell lines. All 5FU-resistant cell lines overexpressed TS mRNA and protein as compared with the parent cell lines. LV enhanced the cytotoxicity of 5FU against three of the four 5FU-resistant cell lines studied (excluding MKN74). To the best of our knowledge, this is the first demonstration of enhancement of the antitumor activity of 5-FU by LV, not only against parent gastric cancer cell lines, but also against the 5FU-resistant cell lines derived from the parent lines.

A previous study reported that a higher concentration of LV added to 5FU formed more TS ternary complex, thereby enhancing the cytotoxicity of 5FU [21], whereas only limited enhancement of 5FU cytotoxicity was obtained at a fixed concentration of LV [19]. In the present study, MKN74/5FU and NCI-N87/5FU were sensitive only to 5FU concentrations lower than approximately 10 μM. These results suggest that lower concentrations of LV may be inadequate for the formation of TS ternary complex.

It is interesting that the sensitivity of the parent and 5FU-resistant lines of MKN74 to 5FU cytotoxicity was not enhanced by LV. There are two likely explanations for this finding. First, in vitro as well as in vivo experiments have shown than the enhancement of 5FU cytotoxicity by LV is greater in cell lines with relatively high TS expression [15, 19]. Because both the parent and 5FU-resistant lines of MKN74 showed low expression levels of TS mRNA (Fig. 2a), LV could not enhance the cytotoxicity of 5FU against these lines. Second, the enhancement of 5FU cytotoxicity against these lines may be saturated by inherent folic acid contained in the cell culture medium and serum because their TS expression was limited (Fig. 2a). For this reason, the addition of LV did not appreciably enhance 5FU cytotoxicity.

Gene expression analysis in previous studies showed a 7-fold increase in TS mRNA in colorectal cancer cell lines resistant to 5-fluoro-2′-deoxyuridine (FdUrd), an intermediate metabolite of 5FU (DLD-1/FdUrd). On average, TS mRNA expression in two 5FU-resistant colorectal cancer cell lines (DLD-1/5FU, HT-29/5FU) and one gastric cancer cell line (NUGC-3/5FU) was 1.6 fold higher than that of the parent cells on Northern hybridization assay [13, 22]. Furthermore, both the TS gene expression level and protein level were significantly associated with response to 5FU-based therapy in human colorectal and gastric tumors [23, 24]. In another study, TS activity was 2- to 3 fold higher in 5FU-resistant colorectal cancer xenografts than in parent cell lines, with no marked change in TP or OPRT activity on in vivo assays [14].

TS enzyme activity has been shown to significantly correlate with 5FU sensitivity in vitro and vivo [7, 25], and correlations among TS copy number, TS mRNA expression level, and drug sensitivity have been demonstrated in several cancer cell lines [26]. A clinical meta-analysis suggested that, as compared with tumors expressing low levels of TS, those that express high levels of TS were associated with poor overall survival in patients with advanced colorectal cancers who received TS inhibitors and with poor progression-free survival in patients who received a variety of treatments in an adjuvant setting [27]. These results suggest that the expression of TS is related to sensitivity to 5FU and that resistance to 5FU might be caused by increased expression levels of TS. Although this study had the same results as previously reported, this is the first report with gastric cancer cells.

Although the mRNA expression of TP had decreased in 5FU-resistant cell lines in this study, this decrease might not have appreciably contributed to acquisition of resistance to 5FU, because the baseline expression levels of TP in parent cells were less than the expression levels of TS (Fig. 2c); the same applies for the mRNA expression of DPD in parent cell lines (Fig. 2b). Although the mRNA expression of OPRT differed only slightly between three 5FU-resistant cell lines and their respective parent cell lines in this study, Tsutani et al. [28] reported underexpression of OPRT mRNA in 5FU-resistant MKN45 cell lines. These results support a correlation between underexpression of OPRT mRNA and the acquisition of 5FU resistance by gastric cell lines.

Collectively, our results suggest that upregulation of TS expression is significantly associated with the acquisition of 5FU resistance by gastric cancer cell lines. These cell lines may be appropriate models for investigating mechanisms underlying the acquisition of resistance to 5FU, because their behavior is consistent with the results of previously studies of cell lines and clinical samples.

Many previous studies reported that co-administration of LV with 5FU improves therapeutic efficacy in mice with colorectal cancer xenografts [29, 30]. Tsujimoto et al. reported that LV increased the antitumor activity of 5FU by 24–32 % against parent and 5FU-resistant colorectal tumor xenografts (KM12C/5FU). Their results suggested that enhancement of the antitumor effect of 5FU by LV was most prominent in 5FU-resistant colorectal tumor xenografts with high TS expression [15]. Spears et al. reported that resistance to 5FU was caused by low FdUMP levels in more than half (53 %) of all colorectal specimens that showed 5FU resistance associated with poor TS inhibition, with 40 % of the specimens showing low FdUMP levels as the sole mechanism for resistance to 5FU [31]. The addition of LV may thus be effective for antitumor chemotherapy against 5FU-resistant cell lines. In our study, the antitumor activity of 5FU was significantly enhanced by LV in three cell lines, excluding MKN74. MKN74 showed relatively low expression of TS in both the parent and respective 5FU-resistant cell line, which may explain the low cytotoxicity of 5FU with LV, suggesting that RNA dysfunction contributes to the antitumor activity of 5FU against MKN74.

Our study had several limitations. TS mRNA and protein overexpression is a major 5FU resistance-inducing factor, and many studies have suggested that irinotecan-, lapatinib-, gefitinib-, and trastuzumab-induced downregulation of TS is responsible, at least in part, for the synergistic antitumor effect produced by these drugs in combination with 5FU. Such combined treatment may also be effective against 5FU-resistant tumors [32–35]. Our study showed that LV also enhanced the antitumor activity of 5FU. However, these results are based on only four cell lines and therefore must be confirmed by in vivo assays and clinical studies. Another limitation is that the results obtained with the four genes studied cannot explain the mechanism responsible for acquisition of 5FU resistance. To identify the genetic characteristics of these resistant cell lines, we are analyzing gene expression profiles from the early establishment of 5FU resistance, using microarray-based technology.

In conclusion, LV enhanced the cytotoxicity of 5FU not only against parent gastric cancer cell lines, but also against 5FU-resistant cell lines, including those with increased TS expression. It was reported that postoperative treatment with 5FU/LV (RPMI) in patients with gastric cancer was noninferior to S-1, which is an oral fluoropyrimidine derivative consisting of tegafur and two modulators. S-1 is a standard treatment for postoperative adjuvant chemotherapy in Japan [36]. Furthermore, a randomized phase II trial is ongoing to compare the response rates of the following three regimens: S-1/LV, S-1/LV/oxaliplatin, and S-1/cisplatin) (JapicCTI-111635). Our results provide some basic rationale of 5FU with LV when these clinical trials are warranted in patients who have recurrent gastric cancer after 5FU-based chemotherapy.
